# Reliable Atom Probe Tomography of Cu Nanoparticles Through Tailored Encapsulation by an Electrodeposited Film

**DOI:** 10.3390/nano15010043

**Published:** 2024-12-30

**Authors:** Aydan Çiçek, Florian Knabl, Maximilian Schiester, Helene Waldl, Lidija D. Rafailović, Michael Tkadletz, Christian Mitterer

**Affiliations:** Department of Materials Science, Montanuniversität Leoben, 8700 Leoben, Austria; aydan.cicek@stud.unileoben.ac.at (A.Ç.); florian.knabl@alumni.unileoben.ac.at (F.K.); maximilian.schiester@unileoben.ac.at (M.S.); helene.waldl@stud.unileoben.ac.at (H.W.); lidija.rafailovic@oeaw.ac.at (L.D.R.); michael.tkadletz@unileoben.ac.at (M.T.)

**Keywords:** atom probe tomography, nanoparticles, electrodeposition, particle encapsulation

## Abstract

Nanoparticles are essential for energy storage, catalysis, and medical applications, emphasizing their accurate chemical characterization. However, atom probe tomography (APT) of nanoparticles sandwiched at the interface between an encapsulating film and a substrate poses difficulties. Poor adhesion at the film-substrate interface can cause specimen fracture during APT, while impurities may introduce additional peaks in the mass spectra. We demonstrate preparing APT specimens with strong adhesion between nanoparticles and film/substrate matrices for successful analysis. Copper nanoparticles were encapsulated at the interface between nickel film and cobalt substrate using electrodeposition. Cobalt and nickel were chosen to match their evaporation fields with copper, minimizing peak overlaps and aiding nanoparticle localization. Copper nanoparticles were deposited via magnetron sputter inert gas condensation with varying deposition times to yield suitable surface coverages, followed by encapsulation with the nickel film. In-plane and cross-plane APT specimens were prepared by femtosecond laser ablation and focused ion beam milling. Longer deposition times resulted in agglomerated nanoparticles as well as pores and voids, causing poor adhesion and specimen failure. In contrast, shorter deposition times provided sufficient surface coverage, ensuring strong adhesion and reducing void formation. This study emphasizes controlled surface coverage for reliable APT analysis, offering insights into nanoparticle chemistry.

## 1. Introduction

In recent years, atom probe tomography (APT) has revolutionized the field of nanomaterial characterization [1,2,3]. APT enables the chemical visualization of nanomaterials in three dimensions even down to atomic-level resolution and the measurement of the elemental composition of all constituents with a few hundred parts per million (ppm) level chemical sensitivity [3,4,5]. These remarkable features made APT a valuable tool for the investigation of nanomaterials and opened new paths for research on the nano-scale [6].

For successful APT analysis of nanoparticles (NPs), specialized specimen preparation techniques are essential [6]. Focused ion beam (FIB) lift-out and subsequent annular FIB milling are commonly utilized techniques for preparing needle-shaped APT specimens [6]. To decrease FIB preparation time and minimize required efforts, femtosecond (fs)-laser ablation is used as a less elaborate alternative approach. This approach eliminates the lift-out process before annular FIB milling [7].

Various approaches have been suggested to prepare APT specimens containing NPs. Among these, electrophoresis was employed for direct deposition of NPs [8,9,10], nanocrystals, and nanowires [11,12] onto the apex of pre-sharpened APT specimens. However, this technique frequently results in low-density regions and void formation, which can lead to insufficient data for detailed quantitative analysis. Even specimen failure during APT measurement may occur when the NPs are only loosely bonded to the tip apex, whereas only well-bonded NPs may enable their comprehensive analysis [6,9]. As an approach towards a more controlled field evaporation process, embedding of NPs by using various methods in a metallic or oxide matrix was proposed, such as Pt film deposition or in-situ melting of fusible alloys during FIB preparation [13,14,15], magnetron sputter deposition [16,17], and pulsed electrodeposition [18]. However, these methods pose different challenges, are restricted to specific nanomaterials, and may not be universally applicable to the particular NP system of interest.

Kim et al. proposed an alternative approach that is based on the electrodeposition of films to encapsulate spatially separated NPs in a Ni or Ni-oxide matrix [19,20,21]. Using this method, NPs are fully encapsulated within a film with uniform morphology and chemical composition, which is more difficult to achieve with physical vapor deposition due to its line-of-sight limitations which often lead to uneven coatings [22,23]. Uneven field evaporation has been reported for C-supported Pt NP samples due to the dissimilar evaporation fields of C and Pt [19]. Thus, to achieve a suitable tip evaporation, it is crucial to ensure a similar evaporation field for both, NPs and encapsulation matrices, as well as strong adhesion of the interfaces between the NPs and the electrodeposited film. To ensure accurate and reliable analysis, three criteria must be taken into account. First, good agreement of the individual evaporation fields of NPs and matrix is essential to guarantee homogenous evaporation during the measurements and eliminate local magnification effects and related reconstruction artifacts [18,19,24]. Second, it is crucial to ensure that NPs are well encapsulated within the matrix, thereby preventing the presence of residual micro- or nano-voids. The presence of such voids may result in local fracture during APT analysis or creates artifacts during data reconstruction [19]. Finally, to avoid mass spectral overlaps, careful selection of materials is essential. Thus, for the present study, Ni, Co, and Cu were chosen due to their closely matched evaporation fields and well-separated isotopic peaks. To ensure the feasibility of the above–mentioned criteria, NPs need to be efficiently encapsulated within the matrix by optimizing the coverage of NPs on the substrate. Furthermore, the proper choice of substrate and encapsulation film materials allows using the interface as an indicator for the NPs sandwiched between them, facilitating reconstruction and enabling precise data evaluation.

This study presents a methodology for preparing APT specimens by encapsulating Cu NPs between Ni film and Co substrate materials, utilizing electrodeposition. Cu NPs were deposited directly onto Co substrates using magnetron sputter inert gas condensation, resulting in different surface coverages. Prior to encapsulation, scanning electron microscopy (SEM) was used to confirm deposition of NPs on the substrates. To accomplish encapsulation of Cu NPs, a suitable electrodeposition process for Ni films onto Co substrates was adapted and optimized. The morphology of the electrodeposited Ni film was investigated by SEM and X-ray diffraction (XRD). Cross-plane and in-plane APT specimens including the encapsulated Cu NPs were prepared using fs-laser processing, followed by annular FIB milling to prepare ready-to-run APT specimens from the pre-sharpened posts. APT analysis was then conducted with varying pulse energies tailored to the respective NP deposition times.

## 2. Materials and Methods

### 2.1. Materials Selection

Co (99.9% purity, HMW Hauner, Röttenbach, Germany) sheets were cut into 5 × 10 × 0.5 mm^3^ pieces using a microPREP PRO FEMTO fs-laser micromachining system (3D-Micromac, Chemnitz, Germany) with a laser wavelength of 532 nm. One side of the substrates was ground up to 4000 grit silicon carbide paper and, subsequently, polished with 3 µm and 1 μm diamond paste. In the final step, the substrates were polished with oxide suspension and immediately thereafter rinsed with isopropanol and washed with tap water. The polished substrates were then sequentially cleaned in an ultrasonic bath for 5 min each with acetone, ethanol, and isopropanol, followed by rinsing with distilled water to remove any remaining solvent residues and drying in hot air. Finally, N_2_ gas was used for pulsed plasma cleaning of the substrates in a Tetra 30 Low-Pressure Plasma System (Diener electronic, Ebhausen, Germany) at 0.2 mbar for 20 min.

Despite the structural dissimilarity, Ni and Co are expected to demonstrate strong adhesion, which combined with their similar evaporation fields ensures compatibility in APT analysis. The non-overlapping Ni, Co, and Cu peaks in the mass spectra makes them reasonable choices as film (Ni) and substrate (Co) materials for the encapsulation of (Cu) NPs.

### 2.2. Cu NP Synthesis and Characterization

**Figure 1:**
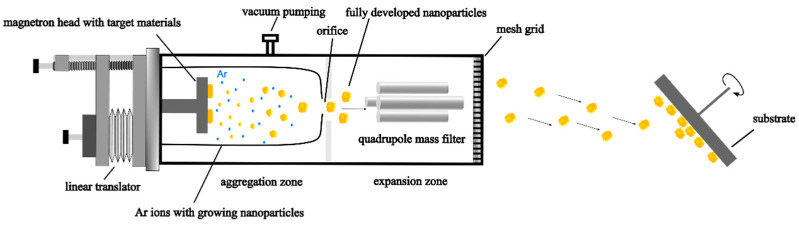
Schematic of NP synthesis by DC magnetron sputter inert gas condensation.

The NP deposition experiments were performed by direct current (DC) magnetron sputter inert gas condensation using a MiniLab 125 vacuum system (Moorfield Nanotechnology, Knutsford, UK) equipped with a NL-UHV nanoparticle source (Nikalyte, Bicester, UK). A schematic illustration of the experimental setup for NP deposition system is depicted in Figure 1.

The NP source itself consists of two components, i.e., the magnetron head and the aggregation zone with the attached quadrupole mass filter (QMF, Nikalyte, Bicester, UK), both with a diameter of 125 mm as detailed in Ref. [25]. The magnetron head is equipped with three water-cooled magnetron cathodes, covered by three metal targets. In this work, NPs were generated from a single Cu target (99.999% purity, Kurt J. Lesker, Dresden, Germany) with a diameter of 25.4 mm and a thickness of 3.2 mm. Before deposition, the base pressure in the deposition chamber was recorded at 6 × 10^−7^ mbar. Ar was introduced as a sputtering gas, maintaining a constant flow rate of 40 sccm. Sputtering was carried out using a constant current of 250 mA and a length of the aggregation zone, where the NPs are formed, of 110 mm. Under the pressure conditions of the aggregation zone, sputtered kinetic Cu atoms form clusters, which grow while travelling through the aggregation zone. The growth process is essentially influenced by two factors; the distance between the target surface and the orifice at the end of the aggregation zone (i.e., the aggregation length, which was set to 110 mm in this work) and the inert gas flow/pressure and eventual composition [26]. NP growth stops due to rapid cooling caused by the pressure difference between aggregation zone (with a pressure around 0.4 mbar, as reported in Refs. [25,27]) and expansion zone (with a pressure of 1 × 10^−3^ mbar). Upon exiting the 3-mm-diameter orifice, it is assumed that NPs have finished their growth and carry a single charge [28,29].

After the orifice, a QMF consisting of two pairs of cylindrical rods is placed. The opposite rods are electrically connected to each other. The QMF allows to select charged NPs based on their mass-to-charge ratio. As NPs are assumed to be single-charged, the QMF may be used for filtering of NP masses. There, an AC voltage V of 250 V, a DC voltage U of 2.5 V, and a U/V ratio of 0.02 was used. Assuming a spherical shape of the NPs and the theoretical density of Cu, the applied QMF scan mode enables recording the NP size distribution prior to their deposition in an NP size range from 1 nm to 20 nm with a fixed step size of 0.1 nm. NP detection was carried out by measuring the grid current at the mesh grid placed at the end of the QMF, yielding a value proportional to the NP flux. A negative bias voltage of −21 V was applied to the grid for the applied different deposition times of 30 min and 2 min, respectively. This facilitates the in-situ quantification of positively charged Cu NPs leaving the NP source. For NP deposition, no additional heating or substrate bias voltage was applied to the substrate holder. Before encapsulation, sufficient NP deposition was verified by SEM using a TESCAN CLARA scanning electron microscope (TESCAN, Brno, Czech Republic).

### 2.3. Encapsulation of Cu NPs by Electrodeposition

A Ni electrodeposition process was used to encapsulate Cu nanoparticles. A typical Watt’s bath consisting of Ni salts NiSO_4_ × 6H_2_O (300 gdm^−3^), NiCl_2_ × 6H_2_O (40 gdm^−3^), and boric acid H_3_BO_3_ (40 gdm^−3^) with additives was used to obtain smooth Ni coatings. The temperature of the Ni bath during deposition was maintained at 328 K, while the pH value was controlled in a range of 4.2 to 4.5. The Co cathode substrate was placed close and parallel to a Ni sheet (99.99+ % purity, HMW Hauner, Röttenbach, Germany) that was used as an anode. The agitation of the electrolyte was maintained at a rotation speed set to 350 rpm, where care was taken not to wash away the NPs from the substrate surface. The electrodeposition was performed galvanostatically, at a constant current density of 50 mA cm^−2^ with a total deposition time set to 1 min to fabricate a dense Ni film with a thickness of at least 600 nm on the Co substrates decorated with Cu NPs.

Six samples, where the Co substrates were decorated with Cu NPs using two different NP deposition times (2 min and 30 min), were prepared by electrodeposition under identical conditions. Two samples from each deposition time were used for cross-sectional SEM analysis. These samples were prepared either with an ArBlade 5000 broad Ar ion beam milling system (Hitachi, Tokyo, Japan) or by cross-sectioning using an Auriga 40 FIB (Zeiss, Oberkochen, Germany).

The remaining electrodeposited film samples were examined using XRD carried out with a D8 Advance diffractometer (Bruker–AXS, Karlsruhe, Germany) operating with CuKα radiation at 40 kV and 40 mA. The measurements were performed in Bragg–Brentano geometry. Diffractograms were measured in the 2θ range of 35° to 105°, at steps of 0.01°, and a time per step of 1.2 s.

### 2.4. APT Specimen Preparation and Measurement

After electrodeposition of the encapsulating film, cross-plane and in-plane APT specimens were prepared. The APT specimens were first fs-laser processed by the already mentioned fs-laser micromachining system. Following the procedure described in Ref. [7], the cutting steps were performed using a laser power of 2.5 W (60 kHz repetition rate, 100 repetitions), while the power was reduced to 50 mW (60 kHz repetition rate, 342 repetitions) for the pre-sharpening steps. In more detail, the 30 min deposition specimen with the Cu NPs encapsulated between the Ni film and the Co substrate was prepared for cross-plane investigation. There, a 3 × 7 mm^2^ coupon was cut and, subsequently, a microtip array with 15 pre-sharpened posts was created by ablating the surface. In contrast, cross-plane and in-plane APT specimens were prepared for the 2 min NP deposition time. While the cross-plane specimen was prepared as described above, the in-plane specimen was prepared in half grid geometry featuring 5 posts [30]. After laser ablation, all specimens were thoroughly cleaned using a tetra CO_2_ snowjet before the cross-plane specimens were mounted onto a Cu spring clip holder (CAMECA Instruments, Madison, WI, USA) and the in-plane specimen on to an Mk II type holder (Microscopy Supplies, Sydney, Australia). Ready-to-run APT specimens were prepared from the pre-sharpened posts by sequential annular FIB milling steps applying Ga^+^ ions at an ion beam current from 30 nA at 30 kV to 27 pA at 2 kV, according to a standard protocol [31].

All APT experiments of Cu NPs encapsulated between Ni film and Co substrate were carried out in a LEAP 5000 XR atom probe (CAMECA Instruments, Madison, WI, USA) in laser-assisted mode at a specimen base temperature of 50 K. For the Cu NPs grown at a deposition time of 30 min, a pulse energy of 60 pJ was used, whereas for those grown for only 2 min, a pulse energy of 100 pJ was applied. All APT measurements were performed at a pulse repetition rate of 250 kHz and a detection rate of 0.5%. The data reconstruction and visualization were conducted using the commercial software IVAS 6.3 provided by CAMECA Instruments.

## 3. Results & Discussion

### 3.1. Cu NP Deposition

**Figure 2:**
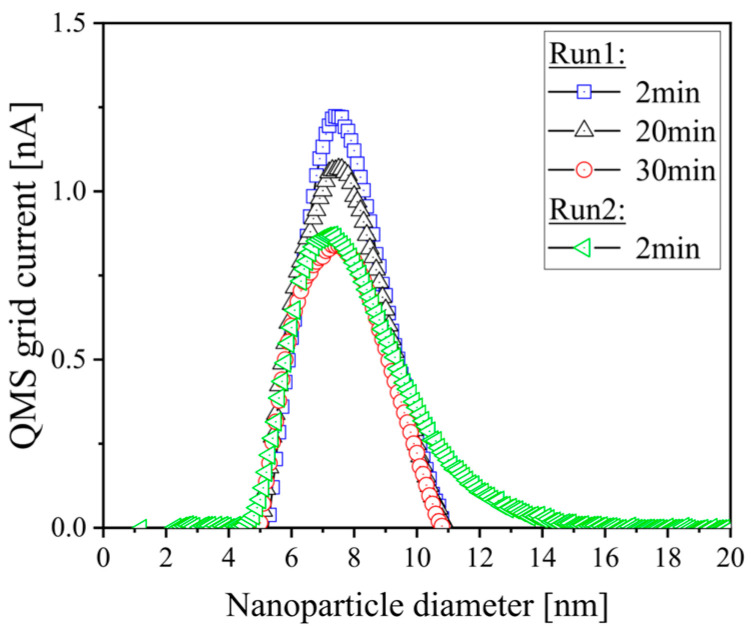
Temporal evolution of the grid current over NP size recorded in-situ during two separate deposition runs using the QMF.

To obtain information about the NP deposition process, the NP size distribution was recorded in-situ for two separate depositions with deposition times of 30 and 2 min, respectively, using the QMF data. The temporal evolution of the measured grid current versus NP size was determined after 2 min, 20 min, and at the end of the 30 min run, as well as at the end of the 2 min run. The obtained data are presented in Figure 2. For the 30 min run, the NP diameter corresponding to the maximum grid current increased slightly from 6.8 nm to 7.3 nm over time. At the end of the short deposition run, a peak NP size of 7.1 nm was obtained. Overall, the NP size distribution changed only slightly over deposition time, with both runs showing similar NP sizes. The observed slight shift can be attributed to the progressing sputter target erosion, leading to a slight increase in NP sizes, a lower NP flux as expressed by the decrease in grid current and a slightly broader size distribution [32].

**Figure 3:**
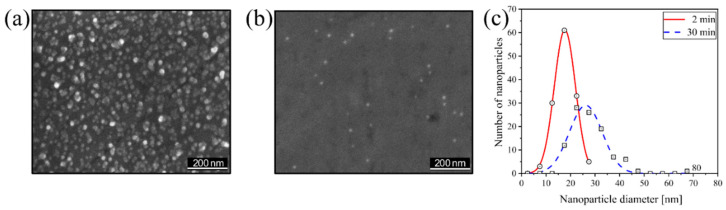
Secondary electron SEM micrographs of Cu NPs on Co substrates after deposition times of (**a**) 30 min and (**b**) 2 min. (**c**) Corresponding size distributions of Cu NPs, where the obtained histograms with a bin width of 5 nm have been fitted with Gaussian functions.

The SEM micrographs in Figure 3a,b reveal as-deposited Cu NPs on Co substrates, where bright spots indicate the deposition of individual or agglomerated NPs. The in-situ QMF data presented in Figure 2 were compared with calculations of the average particle size and surface coverage taken from Figure 3a,b using the open-source software Gwyddion (version 2.63) [33], where all NPs visible in the SEM micrographs were individually selected and analyzed. Figure 3a illustrates a surface fully covered by Cu NPs after a deposition time of 30 min, with the majority of NPs exhibiting diameters in the range of 28 nm ± 8 nm (see Figure 3c). After the 2 min deposition run (Figure 3b), the surface coverage was found to reach 0.9% with an average particle size of 17 nm ± 4 nm given in Figure 3c. The deposited Cu NPs exhibit a random distribution, with those exceeding 15 nm identified as agglomerated NPs, as the in-situ QMF data in Figure 2 indicate NP sizes ranging from 1–15 nm [34]. Moreover, it should be considered that very small and individual NPs could not be observed by SEM due to the inherent resolution limit. Therefore, such small NPs were not detectable and thus could not be taken into account for Figure 3c. In summary, the NP sizes determined by the QMF (see Figure 2) represent a solid basis for the control of the deposition process, whereas the SEM data shown in Figure 3 are more meaningful for the investigation of the properties of an NP-functionalized surface.

### 3.2. Electrodeposited Ni Film

**Figure 4:**
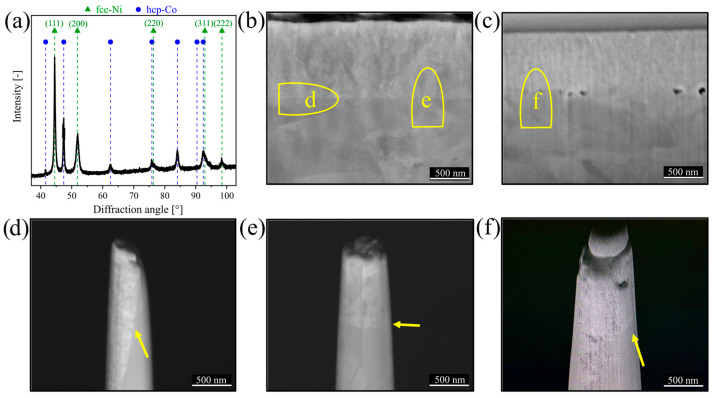
(**a**) X-ray diffractogram for the specimen containing Cu NPs (deposition time 2 min) encapsulated between Ni film and Co substrate. Reference peak positions of fcc-Ni and hcp-Co were taken from Ref. [37]. (**b**) Corresponding SEM image of broad ion beam polished cross-section of the specimen. The regions marked with (**d**,**e**) highlight in-plane and cross-plane specimen preparation for the subsequent APT measurement. (**c**) SEM image of a FIB cross-section with Cu NPs (deposition time 30 min) encapsulated between the Ni film and the Co substrate, where region (**f**) indicates cross-plane APT specimen preparation. Yellow arrows in the pre-prepared APT specimens in (**d**–**f**) mark the interfaces between Co substrate and Ni encapsulation film.

An X-ray diffractogram of the deposited Ni film on the Co substrate is presented in Figure 4a. The standard peak positions for face-centered cubic (fcc)–Ni (ICDD 00-004-0850) and hexagonal close-packed (hcp)–Co (ICDD 01-071-4239) are indicated by dashed lines. Note that the diffractogram does not show distinct peaks of Cu NPs, since their fraction between the Ni encapsulation film and the Co substrate is below the detection limit. The fcc–phase Ni film exhibits strong (111) and weak (200) diffraction peaks. The crystal growth mechanism of Ni by electrodeposition is complex and influenced by multiple factors, with additives playing a crucial role. Factors contributing to texture development include hydrogen evolution and increased electrolyte alkalinity near the Ni-electrolyte interface. These conditions act as interfacial inhibitors during the deposition process [35,36]. The comparison of the intensities measured for the deposited Ni film on Co substrate with the ICDD 00-004-0850 card for fcc–Ni given indicates a slightly preferred (100) growth.

A SEM image of a broad ion beam polished cross-section of Cu NPs deposited for a time of 2 min and encapsulated between Ni film and Co substrate is shown in Figure 4b. The thickness of the Ni film is approximately 900 nm in Figure 4b. For this specimen, the cross-section does not indicate any obvious signs of disruption of the dense and fine-grained film morphology or defects created by the NPs. For the specimen including Cu NPs deposited for a time of 30 min, FIB milling was used to prepare the cross-section, which is shown in Figure 4c. There, some defects such as pores and voids can be found at the interface between the 700 nm thick Ni film and the Co substrate. These defects stem most likely from the high surface coverage of Cu NPs (see Figure 3a), which prevents the full encapsulation of the NPs. In-plane and cross-plane APT specimens, prepared either parallel or perpendicular to the interface for an NP deposition time of 2 min and a cross-plane APT specimen prepared perpendicular to the interface for an NP deposition time of 30 min, are presented in Figure 4d–f, where the NP decorated interfaces are marked by yellow arrows.

### 3.3. Atom Probe Tomography

**Figure 5:**
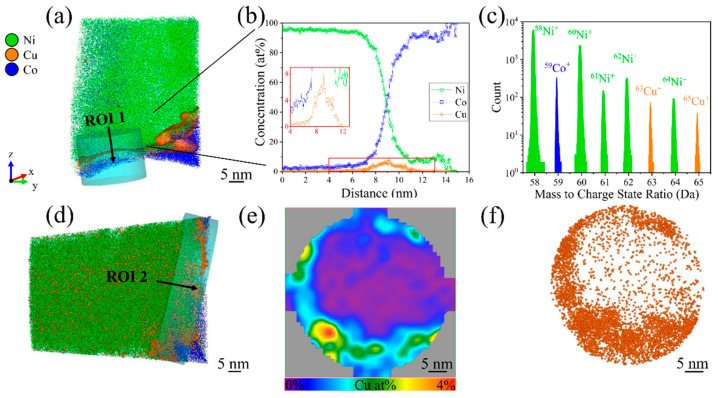
Summary of the outcome of the APT investigation of a cross-plane specimen with Cu NPs (deposition time 30 min) encapsulated between Ni film and Co substrate, with 6 million ions detected during the measurement: (**a**) Clipped 3D APT reconstruction with a cylindrical volume of 20 × 20 × 15 nm^3^ showing the interface in ROI 1, where Cu NPs are encapsulated (the isoconcentration surface of Cu NPs was set at 2 at%). (**b**) Corresponding 1D concentration profiles along the axis of the cylindrical ROI 1 indicated in (**a**). (**c**) Corresponding mass spectrum in the 58–65 Da region of ROI 1. (**d**) Side-view clipped APT reconstruction with a cylindrical volume of 23 × 45 × 45 nm^3^, representing ROI 2 positioned at the fractured interface. (**e**) 2D contour plot of the Cu concentration and (**f**) top view of ROI 2 showing the spatial distribution of Cu atoms towards the z axis of the reconstruction. Note that the spheres shown in (**f**) are used solely for visualization of a fraction of the detected Cu ions and should not be interpreted as individual NPs.

A clipped three-dimensional (3D) atom map of the prepared cross-plane specimen with Cu NPs deposited for 30 min and encapsulated between the Ni film and the Co substrate is presented in Figure 5a. Here, the region of interest (ROI) is defined as cylindrical volume at the NP decorated interface between Ni film and Co substrate. In the APT mass spectrum in Figure 5c, the major peaks could be assigned to single charge states of Ni^+^, Co^+^, and Cu^+^. Additionally, Ni-hydride peaks were detected, which can be attributed to the presence of residual gaseous hydrogen reacting with Ni^+^ during the APT measurement. In addition, as Ni^+^ acts as a non-noble catalyst for hydrogen evolution, it can promote hydrogen incorporation during the growth of the Ni layer in the electrodeposition process [20]. Subsequently, the incorporated hydrogen and its distribution was omitted from any further analysis and the 1D concentration profile shown in Figure 5b is solely based on the ranged mass spectrum and corresponding ion species assigned to the individually observed peaks shown in Figure 5c.

In addition to primary peaks of Ni^+^, Co^+^, and Cu^+^, trace amounts of species such as carbon (0.30 at%), oxygen (0.22 at%), and gallium-related compounds (0.06 at%) were detected. Within these totals, several Ni-based compounds were identified, in particular Ni-oxide (e.g., NiO at 0.22 at%, NiOH at 0.10 at%, NiOC at 0.07 at%), -carbide (e.g., NiC_2_ at 0.02 at%, NiCH at 0.01 at%), and -sulfide based compounds (e.g., NiS at 0.08 at%, NiSO_4_ at 0.01 at%). H_2_0 (0.02 at%) was also detected. Although these species indicate complex chemical interactions at the interface, they were present in minimal concentrations and did not significantly affect the overall concentration profile of the primary peaks. Thus, they were deliberately excluded from the concentration analysis to maintain clarity and focus on the primary peaks central to this study.

The Cu^+^ peak at 63 Da in Figure 5c appears stronger than that at 65 Da, which agrees well with the more pronounced abundance of the isotope ^63^Cu compared to ^65^Cu [20,38,39]. In the 1D concentration profile in Figure 5b, the interface, defined by the gradual transition from Ni^+^ to Co^+^ and highlighted in the red-framed inset, shows an increase in Cu concentration up to 8.8 at%. Shortly after the detection of the first Co^+^ ions, between 10 and 14 nm, the specimen fractured. At this point, the Co concentration was only around 75 at%, while the Ni concentration decreased only to 13.1 at% but does not drop to zero. Instead, Co^+^ and Ni^+^ were simultaneously detected, indicating the potential failure of the APT specimen due to structural instabilities at the interface.

Overall, Figure 5 demonstrates that the careful selection of Ni^+^, Co^+^, and Cu^+^ allows for their successful detection, enabling the identification of Cu NPs at the interface between the Ni film and the Co substrate. However, the high surface coverage of Cu NPs fosters formation of micro- and nano-voids (see Figure 4c). These voids can create areas of weak adhesion, where chemical species like Ni-oxides and -carbides can accumulate or form. A similar behavior has been observed in Ni-based alloys, where voids within the oxide scale act as traps for impurities [40]. The accumulation may result in fracturing and/or delamination at the interface during the APT measurement, as evidenced by the side view of the entire APT specimen in Figure 5d, which indicates that the Co substrate has not been fully reached before fracture of the APT specimen occurred. For the highlighted ROI 2 in Figure 5d, the detailed Cu NP decoration at the interface is depicted in Figure 5e,f, illustrating the local accumulation of Cu NPs. The spatial location of the fracture suggests a weak interface adhesion.

**Figure 6:**
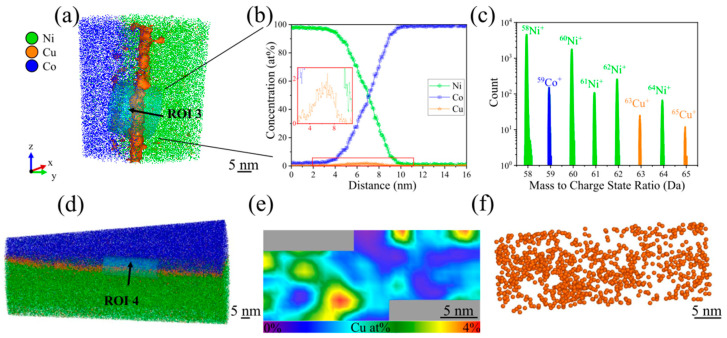
Summary of the outcome of the APT investigation of an in-plane specimen with Cu NPs (deposition time 2 min) encapsulated between Ni film and Co substrate, with 40 million ions detected during the measurement. (**a**) Clipped 3D APT reconstruction with a cylindrical volume of 20 × 20 × 50 nm³ showing the interface in ROI 3, where Cu NPs are encapsulated (the isoconcentration surface of Cu NPs was set at 2 at%). (**b**) Corresponding 1D concentration profiles along the axis of the cylindrical ROI 3 indicated in (**a**). (**c**) Corresponding mass spectrum in the 58–65 Da region of the ROI 3. (**d**) Side-view clipped APT reconstruction with a cylindrical volume of 10 × 10 × 30 nm^3^ representing ROI 4. (**e**) 2D contour plot of the Cu concentration and (**f**) top view of ROI 4 showing the spatial distribution of Cu NPs towards the direction indicated by the black arrow in (**d**). Note that the spheres shown in (**f**) are used solely for visualization of a fraction of the detected Cu ions and should not be interpreted as individual NPs.

To avoid such delamination problems at the interface between substrate and encapsulating film, the NP coverage of Cu NPs on the Co substrate was reduced by shortening the deposition time to 2 min. In addition, the laser pulse energy during the APT measurement was increased from 60 to 100 pJ to avoid eventual field-induced interfacial fracture [41,42]. As a result, the electric field strength at the apex of the specimen is reduced, and thus also the mechanical stress [43]. Reduction of the deposition time effectively minimized the agglomeration of Cu NPs shown in Figure 3a. In addition, to mitigate the risk of APT specimen fracture, an in-plane measurement as schematically illustrated in Figure 4b was conducted. This approach enables to benefit from the mixed field components of the involved elements during the APT measurement (see Figure 6a). The concentration profile of ROI 3, perpendicular to the interface in Figure 6b confirms the gradual transition from Ni^+^ to Co^+^, within a width of around 8 nm. As indicated in the red-framed inset, the maximum Cu concentration at the interface reaches 1.8 at%. In the APT mass spectrum in Figure 6c, carbon (0.14 at%), oxygen (0.14 at%), gallium-related compounds (0.02 at%), along with Ni-oxide (e.g., NiO at 0.01 at%, NiOH at 0.01 at%) and -nitride based compounds (e.g., NiH at 1.40 at%) were detected only in trace amounts. Similar to Figure 5c, H_2_0 was also detected at 0.02 at%. No Ni-carbides or -sulfides were detected. This is attributed to the shorter deposition time of 2 min, which minimized hydrogen incorporation and prevented significant void formation. As a result, the Ni^+^, Co^+^, and Cu^+^ peaks and other trace species were distinctly resolved in the mass spectrum, with minimal interference from oxides, sulfides, nitrides, and other species, allowing for precise compositional analysis.

The intensities of ^63^Cu and ^65^Cu align closely with their natural isotopic abundance [38], thereby confirming the reliability of APT analysis. Figure 6d provides a side view of the entire APT reconstruction highlighting ROI 4, where the Cu NP decoration at the interface is detailed in Figure 6e,f. These figures show a quite uniform distribution of Cu NPs along the interface. The minor local accumulations indicate a still existing agglomeration tendency of NPs. In addition, with the significantly increased laser pulse energy, delamination and fracture during the APT measurement could be avoided.

**Figure 7:**
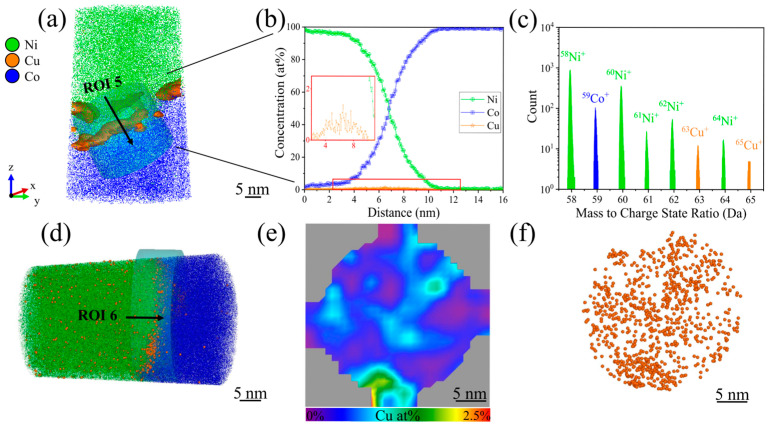
Summary of the outcome of the APT investigation of a cross-plane specimen with Cu NPs (deposition time 2 min) encapsulated between Ni film and Co substrate, with 4.5 million ions detected during the measurement. (**a**) Clipped 3D reconstruction with a cylindrical volume of 20 × 20 × 15 nm³ showing the interface of the frontside of the APT specimen in region of interest 5 (ROI 5), where Cu NPs are encapsulated (the isoconcentration surface of Cu NPs was set at 2 at%). (**b**) Corresponding 1D concentration profiles along the cylindrical ROI 5 indicated in (**a**). (**c**) Corresponding mass spectrum of Cu peaks in the 58–65 Da region of the ROI 5. (**d**) Side-view of the backside of the clipped APT specimen with a cylindrical volume of 10 × 30 × 30 nm^3^ representing ROI 6 positioned at the interface. (**e**) 2D contour plot of the Cu concentration and (**f**) top view of ROI 6 showing the spatial distribution of Cu NPs towards the direction indicated by the black arrow in (**d**). Note that the spheres shown in (**f**) are used solely for visualization of a fraction of the detected Cu ions and should not be interpreted as representing individual NPs.

Given the successful in-plane APT measurement, a cross-plane measurement was also performed to allow a more comprehensive analysis of the interface and the obtained results are summarized in Figure 7. No APT specimen fracture occurred, enabling a thorough analysis of the interface. Figure 7a presents a clipped image of the APT specimen from the very top, i.e., the Ni film, to the bottom, i.e., the Co substrate. The slightly curved shape of the interface, indicating local fluctuations of Cu on the Co substrate at the frontside of the APT specimen, likely stems from APT reconstruction artifacts, arising from combined electrostatic effects due to both the used APT instrument and the specimen shape, described by the k-factor model [5].

The 1D concentration profile along the evaluation axis of ROI 5 in Figure 7b is very similar to Figure 6b and demonstrates again the gradual transition from Ni^+^ and Co^+^ at the interface. The maximum Cu^+^ concentration at the interface reaches 1.30 at%. Figure 7c confirms that the Cu^+^ peaks at 63 Da and 65 Da exhibit again intensities very similar to their natural abundance, similar to the observations in Figure 6c. Minor species, including carbon (0.13 at%), oxygen (0.14 at%), sulfides (0.02 at%), and gallium-related compounds (0.03 at%, stemming from FIB preparation), were detected, along with Ni-oxide based phases (e.g., NiO at 0.03 at%, NiO_2_CH at 0.01 at%). Unlike the results in Figure 6c, Ni-carbides as NiC_2_ (0.01 at%) were present, while Ni-sulfides were identified only in trace amounts. Similar to the observations in Figure 5 and Figure 6, H_2_O was detected at 0.02 at% in Figure 7. Figure 7d provides a view of the entire APT specimen seen from the backside, revealing a smoother interface in ROI 6 without local fluctuations of Cu^+^ compared to Figure 7a. The spatial distribution in Figure 7e,f indicate that the Cu NPs are uniformly spread and not locally accumulated in specific regions.

## 4. Conclusions

We developed a methodology for preparing atom probe tomography (APT) specimens by sandwiching Cu nanoparticles between a Ni encapsulation film grown by electrodeposition and a Co substrate. Ni and Co were selected for their field evaporation properties, which closely align with Cu, and to avoid significant peak overlaps during APT analysis. The Ni-Co interface served as a marker during the measurements and following evaluation, facilitating accurate localization of the Cu nanoparticles.

The deposition of Cu nanoparticles was precisely controlled via magnetron sputter inert gas condensation, with two distinct deposition times set to 30 min and 2 min, respectively, showcasing the effect of surface coverage on preparation and measurement characteristics of APT specimens. A combination of femtosecond-laser ablation and focused ion beam milling was utilized for APT specimen preparation, eliminating the time-consuming focused ion beam lift-out step. Both in-plane and cross-plane APT specimens were prepared. The longer nanoparticle deposition time resulted in larger surface coverage and more agglomerated Cu nanoparticles, which in combination with the low APT pulse energy led to interface failure. Several peaks corresponding to Ni-hydrides, carbides, oxides, and other species were detected at the interface and are assumed to contribute to low interfacial adhesion. Conversely, the shorter deposition time of 2 min, in addition to an increased pulse energy, yielded a sufficient surface coverage, enabling both cross-plane and in-plane specimen to survive the APT measurements.

Our approach to sandwich nanoparticles at the interface between an encapsulation film and a substrate, where the involved materials fulfill requirements in terms of matching evaporation fields and avoidance of peak overlaps, provides a feasible and efficient method for preparing APT specimens. In addition, a suitable nanoparticle surface coverage at the interface and optimized APT pulse energy are crucial for avoiding early failure of APT specimen. Although not explored within this study, the developed preparation methodology should also be useful for other nanoparticle materials, provided that the materials used for the encapsulation film and substrate are carefully chosen, and for nanoparticles deposited by other methods, e.g., wet chemistry. Future work should focus on further refining encapsulation techniques and exploring their applicability to studying the chemistry of a wide range of nanoparticle materials.

## Data Availability

The data that supports this finding are available from the corresponding authors upon reasonable request.
